# Genomic Analysis of Mic1 Reveals a Novel Freshwater Long-Tailed Cyanophage

**DOI:** 10.3389/fmicb.2020.00484

**Published:** 2020-04-08

**Authors:** Feng Yang, Hua Jin, Xiao-Qian Wang, Qiong Li, Jun-Tao Zhang, Ning Cui, Yong-Liang Jiang, Yuxing Chen, Qing-Fa Wu, Cong-Zhao Zhou, Wei-Fang Li

**Affiliations:** Hefei National Laboratory for Physical Sciences at the Microscale and School of Life Sciences, University of Science and Technology of China, Hefei, China

**Keywords:** freshwater cyanosiphophage, Lake Chaohu, genome sequence, terminase large subunit, DNA polymerase γ, evolution

## Abstract

Lake Chaohu, one of the five largest freshwater lakes in China, has been suffering from severe cyanobacterial blooms in the summer for many years. Cyanophages, the viruses that specifically infect cyanobacteria, play a key role in modulating cyanobacterial population, and thus regulate the emergence and decline of cyanobacterial blooms. Here we report a long-tailed cyanophage isolated from Lake Chaohu, termed Mic1, which specifically infects the cyanobacterium *Microcystis aeruginosa*. Mic1 has an icosahedral head of 88 nm in diameter and a long flexible tail of 400 nm. It possesses a circular genome of 92,627 bp, which contains 98 putative open reading frames. Genome sequence analysis enabled us to define a novel terminase large subunit that consists of two types of intein, indicating that the genome packaging of Mic1 is under fine control via posttranslational maturation of the terminase. Moreover, phylogenetic analysis suggested Mic1 and mitochondria share a common evolutionary origin of DNA polymerase γ gene. All together, these findings provided a start-point for investigating the co-evolution of cyanophages and its cyanobacterial hosts.

## Introduction

Cyanobacteria, which have existed on the earth for more than 3.5 billion years, are widely distributed in aquatic environments (Zhang and Gui, 2018). As a group of photosynthetic bacteria, cyanobacteria provide the source of primary production of oxygen, nitrogen, and carbon, and act as a model organism for studying the coordination of carbon and nitrogen metabolisms (Jiang et al., 2018). Beyond contributing to biogeochemical cycle, blooms also yield toxicity and hypoxia of waterbodies, which was first investigated in the Lake Alexandrina of Australia in 1878 (Francis, 1878). From the ecological perspective, blooms are increasing in frequency, magnitude, and duration in recent years, and cause the death of fish and risk to human diet (Paerl et al., 2001; Huisman et al., 2018). Therefore, it becomes imperative to devise an effective strategy to mitigate and control the water blooms.

Cyanophage, the classic companion virus of cyanobacteria, is a key factor that mediates the host communities, food web, carbon cycling, and nutrient recycling. It also has a potential impact on the regulation of cyanobacterial bloom through lysis-induced mortality, metabolic outputs as well as altering diversity and community structures (Zhang and Gui, 2018). Since the first complete genome sequence of cyanophage P60 that infects the marine *Synechococcus* was reported in 2002 (Chen and Lu, 2002), the genomes of 112 cyanophages have been sequenced and deposited in Virus-Host Database.^1^ However, only 14 genomes of freshwater cyanophages have been sequenced. The first one came from the myophage Ma-LMM01 of *Microcystis aeruginosa* in 2008 (Yoshida et al., 2008). Afterward, four contractile-tailed phages, MaMV-DC (Ou et al., 2015a), S-CRM01 (Dreher et al., 2011), A-1 and N-1 (Chenard et al., 2016), five short-tailed phages, PP (Zhou et al., 2013), Pf-WMP3 (Liu et al., 2008), Pf-WMP4 (Liu et al., 2007), A-4L (Ou et al., 2015b) and S-EIV1 (Chenard et al., 2015) and one so-called tailless phage PaV-LD (Gao et al., 2012) were sequentially isolated from different sources of freshwater. Nevertheless, only three complete genomes of freshwater siphophages, S-2L (Marliere, 2006), S-LBS1 (Zhong et al., 2018), and CrV-01T (Martin et al., 2019) have been reported to date.

Lake Chaohu (117°16′54″E-117°51′46″E, 30°25′28″N-31°43′28″N), located at the south of the capital city Hefei of Anhui province, is one of the five largest freshwater lakes in China. It annually suffers from massive water blooms due to the fast growth of cyanobacteria, accompanied with the rapid industrialization and urbanization of the surrounding areas in the past decades. We successfully isolated a freshwater cyanosiphophage Mic1 from Lake Chaohu. Genome sequencing showed that Mic1, which depicts *Microcystis*-specific, possesses a genome of 92,627 bp (GenBank accession No. MN013189), consisting of 98 putative open reading frames (ORFs). Genome sequence analysis indicated that Mic1 contains a hypothetical ParABS plasmid partition system and a prophage antirepressor, suggesting that the prophage of Mic1 might also exist in the host. Moreover, Mic1 encodes a novel terminase large subunit that consists of two types of intein, which might be involved in the fine control of DNA packaging and phage maturation. In addition, Mic1 encodes a mitochondrion-like DNA polymerase gene, which might be transferred from a common ancestor of mitochondrion or its companion phage.

## Materials and Methods

### Isolation and Purification of Cyanophage

**FIGURE 1:**
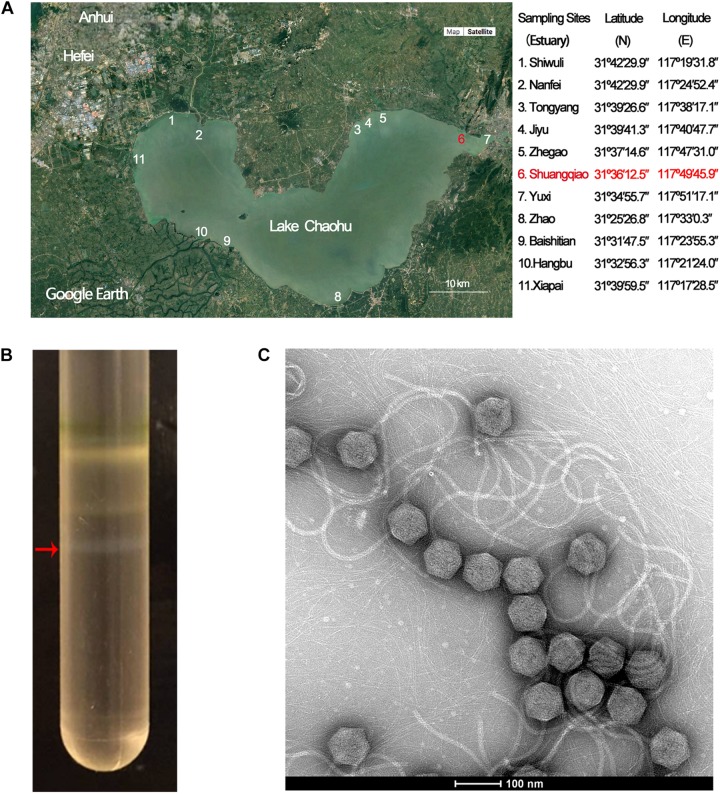
Isolation, purification, and morphological identification of Mic1. **(A)** Location of Lake Chaohu (from the Google Earth) and the coordinates of 11 major river estuaries to Lake Chaohu. The red mark shows the site where Mic1 was isolated. **(B)** CsCl density gradient ultracentrifugation of Mic1. Mic1 concentrated in the fourth layer gradient is indicated by a red arrow. **(C)** Electron micrograph negatively stained Mic1 particles. The cyanophage Mic1 were negatively stained with uranyl acetate and visualized by transmission electron microscopy at 120 kV accelerating voltage. The phages have an isometric head of ∼88 nm in diameter and a long, non-contractile tail of ∼400 nm in length.

Water samples were collected semimonthly from the estuaries of 11 rivers toward Lake Chaohu since 2016 (Figure 1A). After being concentrated to about 100-fold by ultrafiltration, the water samples were applied to infect 11 cyanobacterial strains isolated from Lake Chaohu (data not shown), then the lytic cyanophages infecting previously named *Microcystis wesenbergii* FACHB 1339 were further isolated by the serial dilution method (Yoshida et al., 2006). Notably, the host *Microcystis wesenbergii* FACHB 1339, which was bought from the Freshwater Algae Culture Collection at the Institute of Hydrobiology, Wuhan, should be classified into *Microcystis aeruginosa* (Harke et al., 2016). Then the crude lysate after phage infection was treated with 1 μg/mL DNase I and RNase at 37°C for 1 h. Afterward, NaCl was added to a final concentration of 0.5 M, followed by incubation at 4°C for 1 h. After centrifugation at 8,000 g for 20 min, the cyanophage particles in the supernatant were pooled and incubated with 10% polyethylene glycol 6,000 at 4°C for 10 h. The pellets after centrifugation were resuspended in SM buffer (50 mM Tris, pH 7.5, 10 mM MgSO_4_, 100 mM NaCl), and further purified by ultracentrifugation at 100,000 g for 4 h with a CsCl density gradient. The cyanophage band with the highest opalescence was collected by a syringe and dialyzed against SM buffer. The isolated cyanophage was named Mic1, denoting the first strain of cyanosiphophage toward its host *M. aeruginosa*.

### Transmission Electron Microscopy

The 4 μL suspension of freshly purified cyanophage Mic1 was layered onto a hydrophilized carbon-coated copper grid, and Mic1 particles were negatively stained with 2% uranyl acetate for 1 min. The particles were examined with a Tecnai G2 Spirit BioTWIN 120 kV transmission electron microscope (FEI Company).

### Genomic DNA Extraction

The phage sample in 2 × lysis buffer (20 mM EDTA and 0.5% SDS) was incubated with 50 μg/mL proteinase K at 56°C for 1 h. The phage suspension was sequentially treated with an equal volume of phenol, phenol-chloroform-isoamyl alcohol (25:24:1) and chloroform, respectively. Subsequently, the DNA was precipitated with 0.3 mol/L CH_3_COONa, pH 7.5 and 3-fold volume of ethanol at −80°C for 1 h. The precipitate of phage genomic DNA was washed twice with 70% ethanol and resuspended with sterile water.

### Genome Sequencing

The sequencing library of phage genomic DNA was constructed using TruePrep DNA library Prep Kit V2, and a total of 10 Gb raw data was generated using Illumina Hiseq 2000 platform. The software Velvet (Version 1.2.07) was applied for genome assembly and 12 contigs showing somewhat similarity with known cyanophages at the protein sequence level were chosen for further analysis. The order and orientation of the 12 contigs were determined by PCR and Sanger sequencing. Finally, the assembled genome was verified using DNAMAN (Lynnon Biosoft).

### Genome Annotation and Characterization

The ORFs were predicted using Glimmer (Delcher et al., 1999) and GeneMarkS (Lukashin and Borodovsky, 1998). The translated ORFs were compared with nr protein database in NCBI^2^ using BLASTp program with *e*-values <10^–3^ and the protein hits with the minimal *e*-value in each species were considered to be orthologs. HHpred analysis against the pfamA database was carried out using the default parameters.^3^ Promoters of the genome were predicted by BPROM (LDF > 5).^4^ Multiple-sequence alignment was performed using the Multalin program.^5^ The circular genome map was drawn using CGView.^6^ The cyanophage proteomic tree and genome alignments were conducted with Viptree^7^ (Nishimura et al., 2017). The genome-wide similarity score (SG) cutoff for clustering was set to ≥0.15 (viral genus-level cutoff, where the SG value of 1 stands for two identical genomes and 0 for no detectable high-scoring segment pairs by tBLASTx), according to the previous study (Morimoto et al., 2019). The heatmap was generated using Gegenees (tBLASTx method, accurate parameters-fragment length: 200 bp; step size: 100 bp), and the Splits Tree dendrogram was calculated using the Nexus file exported from Gegenees.

### Mass-Spectrometric Identification of Phage Proteins

The phage proteins separated by 4–12% gradient polyacrylamide gel were analyzed by liquid chromatography/mass spectrometry (LC-MS/MS) using ion-trap mass spectrometer (Thermo LUMOS) and identified by comparing to the protein/peptide sequences deduced from Mic1 genome as previously described (Jin et al., 2019).

## Results and Discussion

### Isolation and Morphology of Mic1

Lake Chaohu has been severely polluted in recent years, owing to a large amount of industrial waste water and domestic sewage flowing from the rivers of Shiwuli, Nanfei, Shuangqiao and Xiapai, which are major rivers to the lake. Accordingly, we chose the estuaries of 11 major rivers entering Lake Chaohu (Figure 1A) to investigate the cyanophages and their hosts. Cyanophage Mic1 was isolated from a surficial water sample collected at Shuangqiao estuary on October 21st, 2017, which was enriched with *Microcystis*-dominated blooms. Host-range assays showed that Mic1 is only able to lyse a strain of *Microcystis aeruginosa* (previously named *M. wesenbergii* FACHB 1339) isolated from Lake Chaohu, indicating that the infectivity of Mic1 is limited to specific cyanobacterial strains.

Purification of Mic1 from the lysate was performed by CsCl density gradient centrifugation, and the fourth opalescence band containing cyanophages was collected (Figure 1B). The purified cyanophage particles were negatively stained for morphological analysis by electron microscopy, revealing that all particles have a siphoviridal morphotype. As shown in Figure 1C, Mic1 has an icosahedral head with a diameter of 88 nm and a flexible and non-contractile tail of 400 nm in length.

### Genome Sequence of Mic1

**FIGURE 2:**
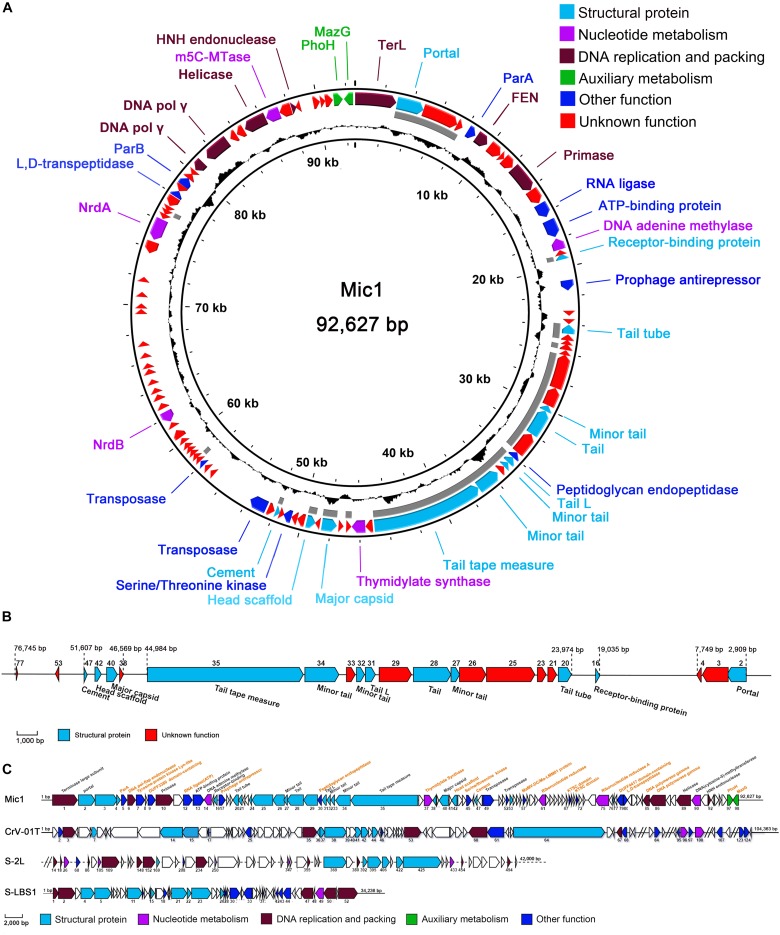
Genomic analyses of Mic1. **(A)** The circular genomic map of Mic1. Circles from the outmost to the innermost correspond to: (I) Predicted ORFs (BLASTp, nr database) with functions are labeled in different colors around the circular map; (II) Gray lines show the structural proteins identified by mass spectrometry; (III) GC content plotted relative to the genomic mean of 35% G + C. **(B)** The structural proteins of Mic1 identified by LC-MS. **(C)** The genomic comparison of four freshwater cyanosiphophages. The unique ORFs of Mic1 are labeled in yellow.

Mic1 has a circular double-stranded DNA genome of 92,627 bp with a G + C content of 35% (Figure 2A). BLASTp and HHpred analyses indicated that Mic1 has 98 ORFs, which encode hypothetical proteins/peptides of 36–2,572 residues in length. Sequence comparison indicated that only 50 hypothetical proteins have homologs, 36 of which have known functions (Supplementary Table 1). These 36 proteins could be divided into five groups: structural proteins, nucleotide metabolism, DNA replication and packing, auxiliary metabolism and others (Figure 2A).

Bioinformatics analyses revealed that the majority of Mic1 structural proteins are similar to those found in other long-tailed phages, for example, the portal protein (ORF2), receptor binding protein (ORF16), phage minor tail (ORF27, ORF32, ORF34), phage tail (ORF28), phage tail L (ORF31), and tail tape measure (ORF35). Moreover, the cryoelectron microscopy (cryo-EM) structure of the icosahedral capsid of Mic1 demonstrated that the major capsid protein and the cement protein are encoded by ORF40 and ORF47, respectively (Jin et al., 2019). To further identify the structural proteins, the purified Mic1 particles were analyzed by mass spectrometry, which identified 23 proteins in total (Jin et al., 2019). As expected, the most abundant proteins are the major capsid protein ORF40 and tail tube protein ORF20. The genes encoding the 23 structural proteins are mainly distributed in three regions (Figure 2B): portal protein ORF2 followed by two proteins ORF3 and ORF4 of unknown function, tail-related proteins containing ORF20 to ORF35 in addition to a downstream receptor-binding protein ORF16, and major capsid ORF40 with accessory proteins (such as the head scaffold and cement). Notably, most of the 23 structural genes are transcribed in the opposite direction against the majority of non-structural genes. In addition, Mic1 harbors two auxiliary metabolic genes, *phoH* (ORF97) and *mazG* (ORF98), the products of which are involved in elevating the phage fitness by altering host metabolism during infection. PhoH belongs to the phosphate regulon that regulates phosphate uptake and metabolism under conditions of low-phosphate and phosphate limitation (Goldsmith et al., 2011), whereas MazG is responsible to reactivate the macromolecular synthesis pathways, via modulating the ppGpp pool, for the propagation of phage progenies in the nutrient-limited cyanobacteria (Bryan et al., 2008; Dreher et al., 2011). Besides, Mic1 encodes nine hypothetical proteins, which share no sequence similarity to any previously identified proteins (Supplementary Table 1 and Figure 2A).

Comparison with the genomes of three freshwater cyanosiphophages CrV-01T, S-LBS1 and S-2L, which are of 104,262, 34,236, and 42,000 bp, respectively, Mic1 possesses genome of 92,627 bp. Despite sharing a similar genome architecture, Mic1 encodes 22 unique components, including ParA (ORF5), ParB (ORF82), head scaffold (ORF42), cement (ORF47), and DNA polymerase γ (ORF85 and ORF86) (Figure 2C).

**FIGURE 3:**
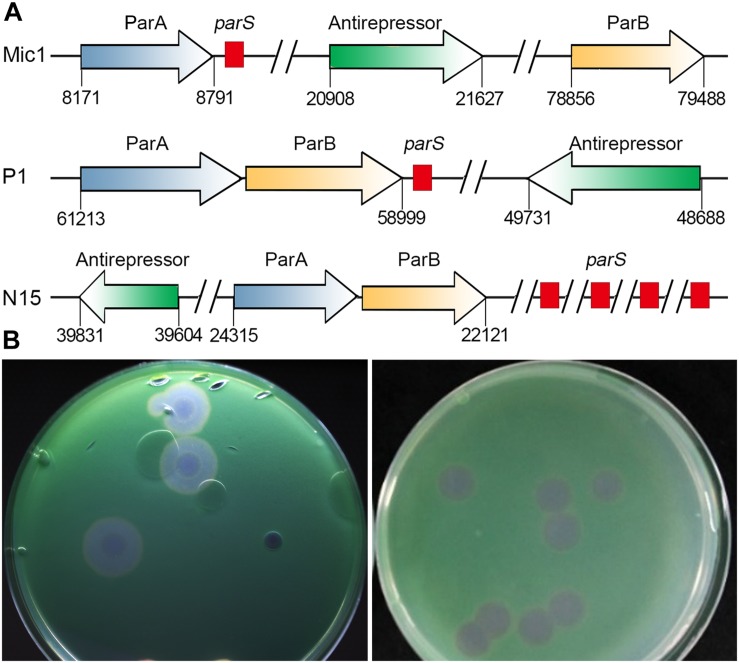
Analyses of Mic1 ParABS system. **(A)** Partitioning loci location in Phages. Genes that encode ParA are depicted by blue arrows, ParB by yellow arrows, the sequences of *parS* are depicted by red box. Genes that encode antirepressor are depicted by green arrows. **(B)** The phage plaques of Mic1. Plaques in the left dish are at lysogenic status, featured with cloudy circular plaques with a bull’s eye at the center, whereas the clear plaques in the right dish are at lytic status.

Notably, we presented here the first report of a cyanophage encoding ParA and ParB, which are involved in DNA partition. ParA and ParB were reported to exist in the low-copy-number phage-plasmids, such as *Escherichia coli* bacteriophage P1 (Vecchiarelli et al., 2010) and N15 (Ravin, 2011). Moreover, a palindromic sequence (_8828_AAATCACCTAAGTTAGGTGATTT_8850_) was also found at the downstream of *parA* gene in Mic1 genome (Figure 3), similar to the previously reported *parS* partition site at the upstream or downstream of *parAB* operon (Brooks and Hwang, 2017). Identification of ParA/B homologs combined with the *parS*-like site indicated that Mic1 might also adopt the ParABS plasmid partition system in the lysogenic cycle, similar to that of phage P1 (Vecchiarelli et al., 2010) and N15 (Ravin, 2011). Moreover, we found that ORF17 of Mic1 most likely encodes a prophage antirepressor (Figure 3A), which was proposed to mediate the lytic induction among temperate phages in the order Caudovirale (Kim and Ryu, 2013). In fact, the phenomena of the bull’s-eyed plaques were found when amplifying Mic1 (Figure 3B). However, the defined condition that switches the lysis–lysogeny transition remains unknown, despite the lysogenic state is most likely favored at the conditions such as low productivity of host cells, poor nutrients, exposure to high temperature or UV (Howard-Varona et al., 2017). Altogether, identification of the ParABS plasmid partition system and the prophage antirepressor strongly indicated that Mic1 might also have a lysogenic cycle at some yet unknown conditions.

**FIGURE 4:**
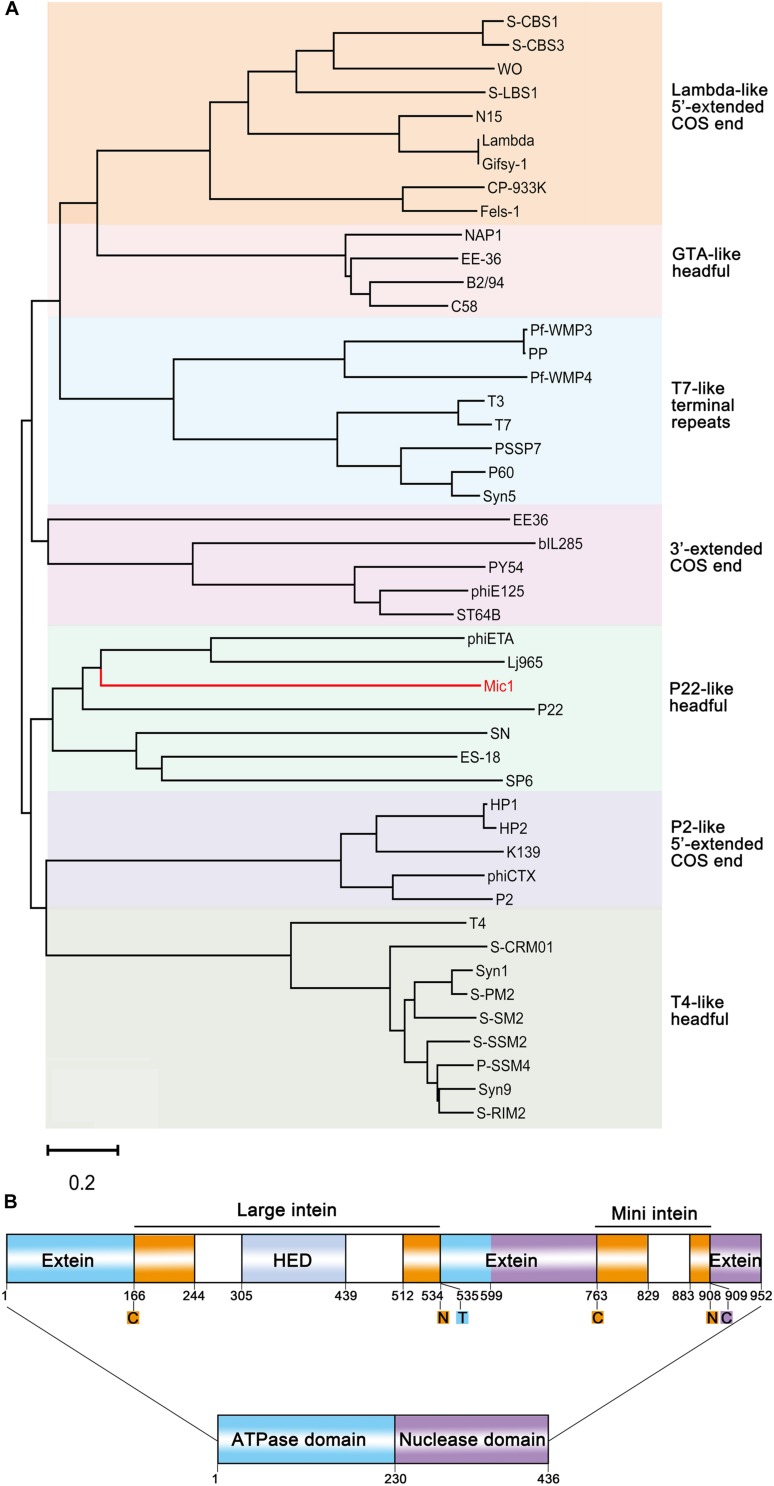
Analyses of Mic1 TerL. **(A)** Phylogenetic tree of TerL proteins from different phages inferred by Neighbor Joining. Colored boxes indicate the characterized clusters which have similar DNA ends. The sequences, except that of Mic1, were classified as described previously (Casjens and Gilcrease, 2009; Huang et al., 2012), and bootstrap analysis was performed with 1,000 repetitions. The scale bar represents 0.2 changes per amino acid. **(B)** Domain organization of TerL. ATPase domain and nuclease domain are indicated by blue and purple box, respectively. The termini of intein are indicated by orange boxes, and the homing endonuclease domain (HED) in the large intein is indicated by the light gray box. Color blocks indicate the conserved amino acid residues at split sites, the oranges (cysteine, asparagine) for the boundary of intein, the blue (threonine) and purple (cysteine) for the boundary of extein.

Phylogenetic analysis based on the large terminase subunit TerL showed that Mic1 falls into the “P22-like headful” cluster (Figure 4A), which has a terminally redundant and circularly permuted genome (Casjens and Gilcrease, 2009). Mic1 is closely related to siphovirus *Lactobacillus thermophilus* prophage Lj964 (Desiere et al., 2000) and *Staphylococcus aureus* phage phiETA (Yamaguchi et al., 2000) based on the pac-site DNA packaging and long tail morphogenesis modules, but distinct from the λ-like freshwater cyanosiphophage S-LBS1 (Zhong et al., 2018).

Notably, TerL of Mic1 contains two different inteins, which are inserted in the ATPase domain and the nuclease domain, respectively; and moreover, the large intein has a homing endonuclease domain (HED) (Figure 4B). Inteins are often mobile, with the ability to post-translationally excise themselves out of the precursor proteins (Perler et al., 1994), usually triggered by external stimuli, such as reactive oxygen species and reactive nitrogen species (Topilina et al., 2015a), reducing agent (Callahan et al., 2011), high temperature (Topilina et al., 2015b), low pH (Wood et al., 1999), high concentration of salt (Reitter et al., 2016), or DNA damage (Lennon et al., 2016). Thus inteins are thought to be biosensors that allow instantaneous splicing to produce the activated proteins. We proposed that the two inteins in the TerL precursor might act as biosensors to control the DNA packaging and maturation of Mic1.

Sequence analysis showed that Mic1 genome has some highly repetitive sequences in the non-coding region. For example, the motif.

GGRYATAT(A)ACAWWTCTAAAAAAAGATGACATAATG GTAACATAAAGAAAAACACAGG that contains the predicted promoter (−35 box TATACA ∼−10 box TGACATAAT) repeats 10 times in the region from 66,031 to 71,646. Another motif GTGCCAGTTYATAAAGTGTCACAATTAGAYTTGACAAAA AGCAAAGTTTATGCAGGC(G)ATAAT containing the predicted promoter (−35 box TTGACA ∼−10 box AGGCAT AAT) repeats 8 times in the region from 67,047 to 69,344. However, more transcriptomic investigations are needed to elucidate the putative multiple promoters that drive the efficient transcription in the lytic cycle of Mic1. In addition, a short repetitive sequence ATCAGTT repeats 20 times in the region from 59,828 to 60,196. In fact, this 7 nt repetitive unit is also widespread in the repetitive regions (ranging from 2 to 40 repetitive units) of *Microcystis aeruginosa* NIES-843 genome. It suggested that this short repetitive unit of Mic1 might come from the host genome through horizontal gene transfer, despite its function in the cyanophage or host remains unknown.

### Evolutionary Analyses at the Genome Level

**FIGURE 5:**
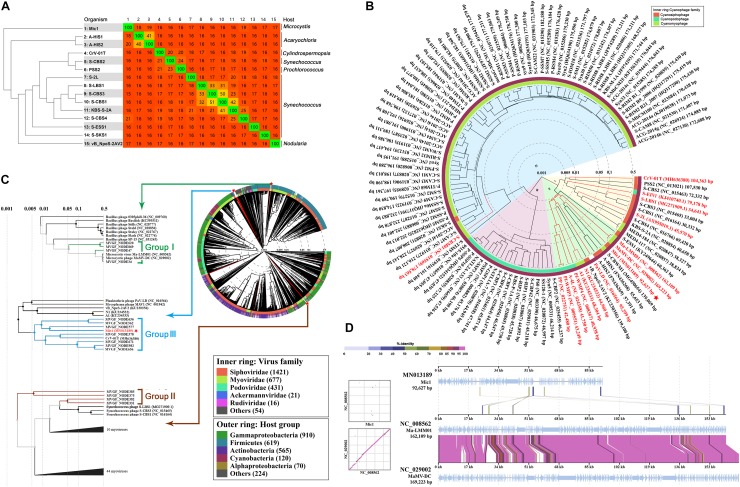
The evolutionary analyses of Mic1. **(A)** Heatmap and phylogenomic tree analyses of cyanosiphophages. The heatmap was generated using Gegenees (tBLASTx method, accurate parameters-fragment length: 200 bp, step size: 100 bp). The numbers show the percentage similarity between the conserved regions of the genomes, whereas the colors reflect the similarity ranging from low (red) to high (green). The Splits Tree dendrogram at the left was calculated using the Nexus file exported from Gegenees. **(B)** The proteomic tree was constructed based on the complete genome sequences of 112 cyanophages. The genome sequences of 12 phages (Mic1, S-2L, S-LBS1, KBS-S-2A, S-EIVI, CrV-01T, A1, N1, S-CBWM1, S-ESSI, A-HIS1, and A-HIS2 were obtained from GenBank and added into ViPTree. The other 100 genome sequences were obtained from Virus-Host Database (https://www.genome.jp/virushostdb/). Cyanophages in the four clusters are shaded in different colors: (I) marine siphophages (yellow), (II) freshwater cyanophages (green), (II) marine podophages (pink), (IV) marine myophages (blue). The previously reported freshwater cyanophages are marked in red, and Mic1 is labeled with a red star. **(C)** Proteomic tree of the complete genome sequences of 2697 dsDNA phages and 15 *Microcystis* viral genomic fragments (MVGFs). Mic1 is labeled with a red star. The whole proteomic tree was generated by ViPTree server ver.1.9. Branch lengths were logarithmically scaled from the root of the entire proteomic tree. **(D)** Genome alignment of Mic1, Ma-LMM01, and MaMV-DC. All tBLASTx alignments are represented by colored lines between two genomes. Color scale represents the tBLASTx percent identity.

Before Mic1, only 14 cyanosiphophages have been genome-sequenced, including 11 marine and 3 freshwater cyanophages. To determine the degree of genomic variability between the cyanosiphophages, the heatmap was clustered based on Bray-Curtis similarity (Zhong et al., 2018) and compared the gene similarities between two phages (Figure 5A). The cyanophages A-HIS1 and A-HIS2 of the unicellular cyanobacterium *Acaryochloris marina* (Chan et al., 2011), CrV-01T of the filamentous *Cylindrospermopsis (Raphidiopsis) raciborskii* (Martin et al., 2019), vB_NpeS-2AV2 of the filamentous nitrogen fixing cyanobacterium *Nodularia* sp. (Coloma et al., 2017), PSS2 of *Prochlorococcus (Sullivan et al., 2009)* were clustered according to their hosts, respectively. In contrast, the two fresh water cyanophage S-LBS1, S-2L were clustered with six marine siphophages S-CBS (1,3,4), KBS-S-2A, S-ESS1, S-SKS1, most likely because they all infect similar hosts of unicellular cyanobacterium *Synechococcus* (Figure 5A). Notably, Mic1, which infects the abundant freshwater unicellular cyanobacteria in the genera *Microcystis*, is distinct from the 14 previously sequenced cyanosiphophages and classified in a unique category. These results implied that cyanosiphophages infecting different host cyanobacteria might possess different genetic lineages.

To further define the relationship between Mic1 and other known cyanophages, proteomic tree was constructed using Viptree based on the genomes of 112 completely sequenced cyanophages from Virus-Host Database (Figure 5B). The cyanophages were grouped into four clusters based on the genome similarity scores (SG): the cyanosiphophages, cyanopodophages and cyanomyophages from marine belong to the clusters I, III, and IV, respectively, whereas most of the freshwater cyanophages including Mic1 fall into cluster II. Despite there is a clear boundary between cluster I and II, some freshwater cyanophages and marine cyanosiphophages are chimerically distributed in clusters I and II, respectively. For example, four freshwater cyanophages CrV-01T, S-EIV1, S-LBS1, and S-2L were grouped into the cluster I, mainly due to homology in tail components or nucleotide synthesis enzymes with their neighboring marine cyanosiphophages. A fine proteomic comparison of cyanophages in cluster II revealed that Mic1 shares similar DNA replication components with A-HIS1, A-HIS2, and S-CBWM1, such as DNA polymerase γ, flap endonuclease (FEN, also known as 5′ nuclease, ORF6), DNA helicase (ORF89) and primase (ORF10). Moreover, Mic1 encodes a DNA polymerase γ with a relatively high similarity to those of A-HIS1, A-HIS2, and S-CBWM1 up to 29–32% sequence identity.

To have a global view of the evolutionary position of Mic1 among the phages, we constructed the proteomic tree (Figure 5C) with the reference genomes of 2,697 phages, in addition to 15 *Microcystis* viral genomic fragments (MVGFs) from Hirosawaniike pond in Japan recently sequenced by metagenomic approaches (Morimoto et al., 2019), using Viptree as previously described (Nishimura et al., 2017). It also indicated that Mic1 possesses a genome distinct from other phages. Moreover, the *Microcystis* cyanophage genomes and the 15 MVGFs could be classified into three groups; and Mic1 falls into group III, the cyanophages of which were reported to have a narrow-host-range that infect highly abundant hosts (Morimoto et al., 2019). It is also consistent with the specificity of Mic1 toward *M. aeruginosa* FACHB 1339, which is one of the most dominant bloom-forming cyanobacterial species in Lake Chaohu.

Despite falling into different groups as shown in Figure 5C, the three genome-sequenced *M. aeruginosa* cyanophages Mic1, Ma-LMM01, and MaMV-DC showed a relatively high similarity at the genome level (Figure 5B). Sequence alignment of the genomes of these three phages also revealed high homology in several coding regions (Figure 5D), especially those corresponding to four Mic1 proteins: ORF45 (Serine/Threonine kinase), ORF49 (transposase), ORF97 (PhoH), and ORF57 (hypothetical protein) (Table 1).

**TABLE 1:** Homologs in cyanophages that infect *M. aeruginosa.*

Mic1	Function	Homologs in Ma-LMM01 (Yoshida et al., 2008)		Homologs in MaMV-DC (Ou et al., 2015a)	
ORF		ORF	Identity (%)	ORF	Identity (%)
45	Serine/Threonine kinase	25	58.24	40	59.34
49	Transposase	31	39.44	127	32.47
97	PhoH	183	37.32	169	37.32
57	Hypothetical protein	158	64.22	145	73.39

### Mic1 Encodes a DNA Polymerase γ

**FIGURE 6:**
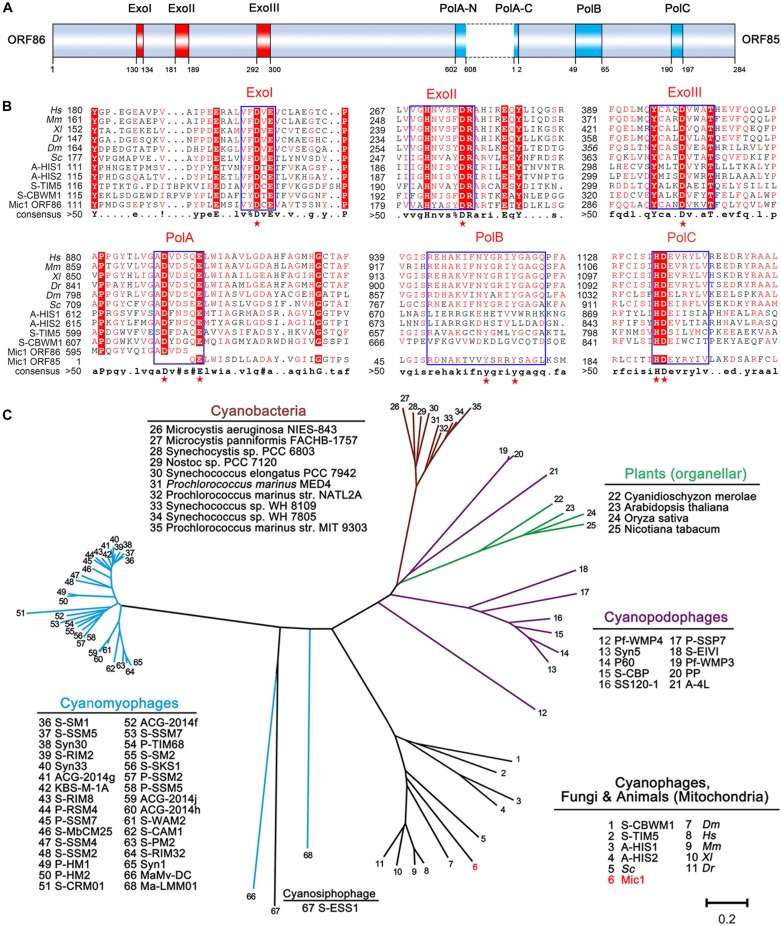
Analyses of DNA polymerase γ. **(A)** Domain organization of Mic1 DNA polymerase γ. The red and blue boxes indicate the conserved exonuclease motifs I–III and polymerase motifs A–C of DNA polymerase γ, respectively. The dotted box indicates the intron between ORF85 and ORF86. **(B)** Multiple-sequence alignment of the conserved DNA polymerase γ from different species. Boxes frame the sequences that are highly conserved, and the key conserved amino acid residues in the exonuclease and polymerase domains are depicted by red stars. The abbreviations are *Hs*, *Homo sapiens*; *Mm*, *Mus musculus*; *Xl*, *Xenopus laevis*; *Dr*, *Danio rerio*; *Dm*, *Drosophila melanogaster*; *Sc*, *Saccharomyces cerevisiae*; A-HIS1 and A-HIS2, *Acaryochloris marina* siphovirus; S-TIM5, *Synechococcus marina* myovirus, S-CBWM1, *Synechococcus marina* myovirus. **(C)** Phylogenetic relationship of DNA polymerases among cyanophages, cyanobacteria and plant organelles. Unrooted phylogenetic tree was constructed based on the neighbor-joining method. The scale bar represents 0.2 changes per amino acid.

DNA polymerase γ, which is specifically localized in the mitochondria, is responsible for the replication of mitochondrial DNA in eukaryotes (Bolden et al., 1977). Despite mitochondria were proposed to be derived from α-proteobacteria, to date no DNA polymerase γ gene has ever been found in any bacteria (Chan et al., 2011). Since 2011, the DNA polymerase γ gene was found in a couple of marine cyanophages, including cyanosiphophages A-HIS1 and A-HIS2 of *Acaryochloris* (Chan et al., 2011), cyanomyophages S-TIM5 (Sabehi et al., 2012), and S-CBWM1 (Xu et al., 2018) of *Synechococcus*. Here we reported a DNA polymerase γ gene in the freshwater cyanophage. The DNA polymerase γ of Mic1 is encoded by ORF85 and ORF86, with an intron of 298 bp between the two ORFs (Figure 6A). Similar to the motif organization of DNA polymerase γ in the model eukaryotes and previously reported cyanophages, ORF86 encodes ExoI∼III motifs and N-terminal moiety of PolA, and ORF85 encodes C-terminal moiety of PolA, PolB, and PolC motifs (Figure 6B). Notably, like the mitochondrial DNA polymerase γ, Mic1 and S-TIM5 possess a PolB motif with a conserved tyrosine, which is absent in A-HIS1/A-HIS2 and S-CBWM1.

To date, the evolutionary origin of mitochondrial DNA polymerase remains unclear. Filee et al. (2002) hypothesized that the replication apparatus of mitochondrial DNA polymerase γ gene was originated from phage, and replaced that of bacterial ancestor. However, the homologs of DNA polymerase γ have been only found in cyanophages, but not present in heterotrophic bacteria or their phages by metagenomic recruitment assays (Xu et al., 2018). As shown in the phylogenetic tree (Figure 6C), DNA polymerases γ of Mic1, together with A-HIS1/A-HIS2, S-TIM5, and S-CBWM1, share a common origin with mitochondrial DNA polymerases in opisthokonts (fungi and animals), but distinct from those in photosynthetic eukaryotes, which belong to origin-unknown plant organellar DNA polymerases (Moriyama et al., 2011; Cupp and Nielsen, 2014). Thus we can predict that, with more cyanophage genome sequences and metagenomics data available, more homologs of mitochondrial DNA polymerase γ genes will be identified. All together, we propose that the DNA polymerase γ genes in mitochondria and cyanophages share a common ancestor.

## Data Availability Statement

The raw datasets for this study can be found in GeneBank, accession No. MN013189.

## Author Contributions

W-FL, C-ZZ, and Q-FW conceived, designed, and supervised the project. FY, HJ, X-QW, QL, and J-TZ performed the cyanophage isolation and characterization experiments. FY and Q-FW analyzed the genome data. NC, Y-LJ, and YC provided some good suggestions for the subject. W-FL, C-ZZ, and FY analyzed data and wrote the manuscript. W-FL and C-ZZ revised the manuscript. All authors discussed the data and read the manuscript.

## Conflict of Interest

The authors declare that the research was conducted in the absence of any commercial or financial relationships that could be construed as a potential conflict of interest.

## Footnotes

1 https://www.genome.jp/virushostdb/index/virus/all

2 https://www.ncbi.nlm.nih.gov/

3 https://toolkit.tuebingen.mpg.de/hhpred

4 http://www.softberry.com/berry.phtml?topic=bprom&group=programs&subgroup=gfi

5 http://multalin.toulouse.inra.fr/multalin/

6 http://cgview.ca/

7 https://www.genome.jp/viptree/
