# Supplementary material for: Genomic Analysis of Mic1 Reveals a Novel Freshwater Long-Tailed Cyanophage
Source: Front Microbiol. 2020 Apr 8;11:484. doi: 10.3389/fmicb.2020.00484 (PMC7156551; doi:10.3389/fmicb.2020.00484)
Supplement: Supplementary file 1 [file Table_1.docx]

**Supplementary Table 1**. Predicted protein-coding genes of Mic1

| **ORF** | **Strand** | **Position** | **Length (aa)** | **Mw (kDa)** | **MS** | **Function protein** | **Related phages or microbes** | **E-value^1^** | **Probability^2^** |
| --- | --- | --- | --- | --- | --- | --- | --- | --- | --- |
| 1 | F | 1-2859 | 952 | 107.89 |  | Terminase large subunit^1^ | *Nostoc* phage N1 | 1.00e^-19^ |  |
| 2 | F | 2909-4798 | 629 | 71.40 | + | Portal protein^1^ | *Nostoc* phage N1 | 3.00e^-19^ |  |
| 3 | F | 4785-7325 | 846 | 96.40 | + |  |  |  |  |
| 4 | F | 7318-7749 | 143 | 16.74 | + | Hypothetical protein^1^ | *Microcystis aeruginosa* | 9.00e^-11^ |  |
| 5 | F | 8171-8791 | 206 | 24.05 |  | ParA family protein^1^ | *Microcystis aeruginosa* DA14 | 7.00e^-74^ |  |
| 6 | F | 8900-9802 | 300 | 34.48 |  | Flap endonuclease^1^ | A-HIS1 phage | 3.00e^-36^ |  |
| 7 | F | 9997-10974 | 325 | 36.06 |  |  |  |  |  |
| 8 | F | 11049-11270 | 73 | 8.68 |  |  |  |  |  |
| 9 | F | 11335-12165 | 276 | 31.23 |  | DUF3380 domain^1^ | *Shewanella frigidimarina* | 1.00e^-46^ |  |
| 10 | F | 12231-14180 | 649 | 74.33 |  | DNA primase^1^ | A-HIS2 phage | 3.00e^-34^ |  |
| 11 | F | 14195-15238 | 347 | 39.59 |  |  |  |  |  |
| 12 | F | 15303-16322 | 339 | 38.37 |  | RNA ligase^1^ | *Chitinophaga eiseniae* | 5.00e^-101^ |  |
| 13 | F | 16586-17824 | 412 | 47.02 |  | ATP-binding protein^1^ | *Microcystis aeruginosa* | 0 |  |
| 14 | R | 17935-18744 | 269 | 31.32 |  | DNA adenine methylase^1^ | *Microcystis aeruginosa* | 0 |  |
| 15 | R | 18848-19045 | 65 | 7.17 |  |  |  |  |  |
| 16 | R | 19035-19448 | 137 | 15.40 | + | Receptor-binding protein^2^ |  |  | 0.96 |
| 17 | F | 20908-21627 | 239 | 26.59 |  | Prophage antirepressor^1^ | *Bacteroides uniformis* | 6.00e^-14^ |  |
| 18 | F | 23053-23340 | 95 | 10.66 |  | Hypothetical protein^1^ | *Enterobacter cloacae* | 2.00e^-14^ |  |
| 19 | F | 23632-23913 | 93 | 10.86 |  | Hypothetical protein^1^ | *Enterobacter cloacae* | 5.00e^-08^ |  |
| 20 | R | 23974-24612 | 212 | 22.67 | + | Tail tube^2^ |  |  | 0.39 |
| 21 | R | 24648-25073 | 141 | 16.32 | + |  |  |  |  |
| 22 | R | 25073-25333 | 86 | 10.41 |  |  |  |  |  |
| 23 | R | 25311-25739 | 142 | 15.76 | + |  |  |  |  |
| 24 | R | 25787-26062 | 91 | 9.96 |  |  |  |  |  |
| 25 | R | 26062-28452 | 796 | 88.05 | + |  |  |  |  |
| 26 | R | 28452-29759 | 435 | 48.66 | + |  |  |  |  |
| 27 | R | 29756-30181 | 141 | 15.86 | + | Phage minor tail protein^2^ |  |  | 0.96 |
| 28 | R | 30178-32031 | 617 | 68.99 | + | Phage tail L^1^ | *Mediterranean* phage uvMED | 3.00e^-09^ |  |
| 29 | R | 32043-33638 | 531 | 57.39 | + | Hypothetical protein^1^ | *Calothrix* sp. NIES-2100 | 2.00e^-13^ |  |
| 30 | R | 33640-34089 | 149 | 17.10 |  | Peptidoglycan endopeptidase^1^ | *Polynucleobacter* *Cosmopolitanus* | 2.00e^-04^ |  |
| 31 | R | 34086-34574 | 162 | 18.75 | + | Phage tail L^2^ |  |  | 0.95 |
| 32 | R | 34577-34969 | 130 | 14.90 | + | Phage min tail^2^ |  |  | 0.91 |
| 33 | R | 34979-35407 | 142 | 14.83 | + |  |  |  |  |
| 34 | R | 35584-37260 | 558 | 60.25 | + | Minor tail^1^ | [*Mediterranean* phage uvMED](https://blast.ncbi.nlm.nih.gov/Blast.cgi#alnHdr_787051941) | 3.00e^-03^ |  |
| 35 | R | 37266-44984 | 2572 | 281.24 | + | Tail tape measure^1^ | [*Listeria monocytogenes*](https://blast.ncbi.nlm.nih.gov/Blast.cgi#alnHdr_1486753676) | 0.01 |  |
| 36 | F | 45100-45609 | 169 | 19.56 |  |  |  |  |  |
| 37 | F | 45616-46557 | 313 | 35.56 |  | Thymidylate Synthase^1^ | *Luteitalea pratensis* | 3.00e^-51^ |  |
| 38 | R | 46569-46931 | 120 | 13.94 | + | Hypothetical protein^1^ | *Calothrix* sp. NIES-2100 | 8.00e^-11^ |  |
| 39 | R | 47213-47350 | 36 | 4.26 |  |  |  |  |  |
| 40 | R | 47567-48613 | 348 | 37.85 | + | Major Capsid^3^ |  |  |  |
| 41 | F | 48779-49063 | 94 | 11.10 |  |  |  |  |  |
| 42 | R | 49060-49680 | 206 | 22.74 | + | Head scaffold^2^ |  |  | 0.98 |
| 43 | F | 49824-50354 | 176 | 20.82 |  |  |  |  |  |
| 44 | F | 50347-50775 | 142 | 15.94 |  |  |  |  |  |
| 45 | F | 50768-51343 | 191 | 21.71 |  | Serine/Threonine kinase^1^ | MaMV-DC/Ma-LMM01 phage | 4.00e^-70^ |  |
| 46 | R | 51369-51536 | 55 | 6.18 |  |  |  |  |  |
| 47 | R | 51607-51906 | 99 | 10.20 | + | Cement^3^ |  |  |  |
| 48 | R | 51992-52480 | 162 | 18.98 |  |  |  |  |  |
| 49 | F | 52610-53854 | 414 | 47.66 |  | Transposase^1^ | *Microcystis aeruginosa* | 0 |  |
| 50 | F | 56907-57224 | 105 | 12.14 |  |  |  |  |  |
| 51 | F | 57423-57764 | 113 | 13.05 |  |  |  |  |  |
| 52 | F | 57867-58175 | 102 | 11.9 |  | iSH4-type Transposase^1^ | *Clostridium* sp.CAG813 | 1.00e^-04^ |  |
| 53 | F | 58223-58558 | 111 | 12.79 | + | Hypothetical protein^1^ | *Butyricimonase* | 1.00e^-10^ |  |
| 54 | F | 58539-58871 | 110 | 12.75 |  |  |  |  |  |
| 55 | F | 58881-59174 | 97 | 11.27 |  |  |  |  |  |
| 56 | F | 59238-59435 | 65 | 7.57 |  | Hypothetical protein^1^ | *Microcystis aeruginosa* | 3.00e^-05^ |  |
| 57 | F | 59490-59819 | 109 | 12.44 |  | MaMV-DC protein^1^ | MaMV-DC phage | 8.00e^-49^ |  |
| 58 | F | 59972-60163 | 63 | 7.23 |  | Hypothetical protein^1^ | *Microcystis aeruginosa* | 1.00e^-26^ |  |
| 59 | F | 60330-60911 | 193 | 22.81 |  |  |  |  |  |
| 60 | F | 61185-61547 | 120 | 13.98 |  |  |  |  |  |
| 61 | R | 61615-62385 | 256 | 29.40 |  | Ribonucleotide reductase^1^ | *Synechococcus* phage S-CBP1 | 3.00e^-42^ |  |
| 62 | F | 62647-63045 | 132 | 15.39 |  | Hypothetical protein^1^ | *Burkholderia pseudomallei* | 2.00e^-15^ |  |
| 63 | F | 63304-63699 | 131 | 15.09 |  |  |  |  |  |
| 64 | F | 63819-64181 | 120 | 13.99 |  |  |  |  |  |
| 65 | F | 64459-64812 | 117 | 13.66 |  |  |  |  |  |
| 66 | F | 65182-65559 | 125 | 14.48 |  |  |  |  |  |
| 67 | F | 65699-65878 | 57 | 6.59 |  |  |  |  |  |
| 68 | F | 66403-66651 | 82 | 9.41 |  | KTSC domain^1^ | *Nocardia* sp. NRRL S-836 | 9.00e^-13^ |  |
| 69 | F | 67243-67419 | 58 | 6.52 |  |  |  |  |  |
| 70 | F | 69468-69644 | 58 | 6.52 |  |  |  |  |  |
| 71 | F | 69818-70066 | 82 | 9.23 |  | KTSC domain^1^ | *Nocardia* sp. NRRL S-836 | 9.00e^-13^ |  |
| 72 | F | 70563-70910 | 115 | 13.42 |  |  |  |  |  |
| 73 | F | 71656-71901 | 81 | 9.47 |  |  |  |  |  |
| 74 | R | 73616-74455 | 279 | 31.98 |  |  |  |  |  |
| 75 | R | 74598-76109 | 504 | 55.69 |  | Ribonucleotide reductase^1^ | S-CBS4 phage | 3.00e^-118^ |  |
| 76 | R | 76160-76525 | 121 | 13.93 |  |  |  |  |  |
| 77 | R | 76608-76745 | 45 | 5.03 | + |  |  |  |  |
| 78 | R | 76811-77107 | 98 | 10.84 |  |  |  |  |  |
| 79 | R | 77175-77879 | 234 | 26.53 |  | DUF4417 domain^1^ | *Tolypothrix* sp.NIES-4075 | 4.00e^-49^ |  |
| 80 | F | 77930-78394 | 154 | 17.28 |  | L,D-transpeptidase^3^ |  |  |  |
| 81 | R | 78374-78841 | 155 | 17.66 |  |  |  |  |  |
| 82 | F | 78856-79488 | 210 | 24.14 |  | ParB |  |  | 0.29 |
| 83 | F | 79460-79882 | 140 | 15.86 |  |  |  |  |  |
| 84 | R | 79879-80094 | 71 | 8.16 |  |  |  |  |  |
| 85 | R | 80222-80959 | 245 | 27.58 |  | DNA polymerase gamma^1^ | *Capsaspora owczarzaki* | 3.00e^-66^ |  |
| 86 | R | 81372-83198 | 608 | 68.51 |  | DNA polymerase gamma^1^ | *Basidiobolus meristosporus* | 4.00e^-133^ |  |
| 87 | R | 83453-83950 | 165 | 19.01 |  |  |  |  |  |
| 88 | R | 83998-84570 | 190 | 22.42 |  |  |  |  |  |
| 89 | R | 84665-86272 | 535 | 60.89 |  | DNA helicase^1^ | *Gluconobacter morbifer* | 3.00e^-21^ |  |
| 90 | R | 86300-87304 | 334 | 38.08 |  | m5C-MTase^1^ | *Microcystis aeruginosa* | 1.00e^-130^ |  |
| 91 | R | 87301-88143 | 280 | 32.80 |  |  |  |  |  |
| 92 | F | 88222-88503 | 93 | 10.95 |  | HNH endonuclease^1^ | *Bacillus licheniformis* | 2.00e^-05^ |  |
| 93 | R | 88500-88766 | 88 | 9.98 |  |  |  |  |  |
| 94 | F | 89720-90202 | 160 | 17.91 |  |  |  |  |  |
| 95 | F | 90280-90513 | 77 | 8.77 |  |  |  |  |  |
| 96 | F | 90555-91097 | 180 | 20.47 |  |  |  |  |  |
| 97 | F | 91147-91785 | 212 | 23.24 |  | PhoH^1^ | Ma-LMM01 phage | 6.00e^-33^ |  |
| 98 | R | 91852-92484 | 210 | 23.24 |  | MazG^1^ | *Arthrobacter* phage Anansi | 3.00e^-09^ |  |

ORF number, gene orientation (F-Forward strand, R-Reverse strand), gene position, molecular weight (Mw) and protein size are noted for each predicted protein-coding gene, and the related phages or microbes are listed for that genes are similar to Mic1.

^1^ predicted by BLASTp program running against the NCBI nr database.

^2^ predicted by HHpred tool.

^3^ solved by cryo-electron microscopy or X-ray.

+ identified by mass spectrometry.
